# “Here, Let Me Do It for You”: Psychological Consequences of Receiving Direct and Indirect Help in Childhood

**DOI:** 10.1111/cdev.14259

**Published:** 2025-06-03

**Authors:** Jellie Sierksma, Eddie Brummelman

**Affiliations:** ^1^ Utrecht University Utrecht the Netherlands; ^2^ University of Amsterdam Amsterdam the Netherlands

**Keywords:** feedback, motivation, self‐perceived ability, unsolicited help

## Abstract

What are the psychological consequences of receiving direct and indirect help in childhood? We conducted three preregistered experiments (*N* = 619, 7–9 years, 80% Dutch, 51% girls, 49% boys, mostly higher socioeconomic status) in the Netherlands (July 2020–July 2022). Children received direct help (correct answer), indirect help (hint), or no help. An internal meta‐analysis showed that children who received help felt less competent, liked the task less, and felt more in need of help. Children who received help also sought fewer challenges (Study 3). Effect sizes were modest. Direct and indirect help had largely similar effects, except that children disliked and misreported receiving direct help more. Thus, despite being well‐intentioned, direct and indirect help can be discouraging.

Imagine a 7‐year‐old child trying their best to solve a puzzle. An adult steps in to offer help: “Here, let me do it for you!” How will the child respond? Will they find the help encouraging, or will they see it as a sign of low ability and withdraw from the puzzle? Undoubtedly, receiving help can benefit children's learning, especially when children struggle or solicit help (Zimmerman 2002). However, adults often provide unsolicited help (Leonard et al. 2021). Such help can be either direct (e.g., giving the right answer) or indirect (e.g., providing a hint). We theorized that unsolicited help, especially if provided in direct ways, may lead children to feel less competent, like the task less, perceive the task as more difficult, and become less motivated. We conducted three preregistered experiments with 7‐to‐9‐year‐old children to test the psychological impact of direct and indirect help.

## Psychological Theory

What are the psychological consequences of receiving unsolicited help? Several theories speak to this question. Self‐determination theory (Ryan and Deci 2000) holds that children have basic psychological needs—competence, autonomy, and relatedness. When these needs are met, children experience greater well‐being and intrinsic motivation. Children might perceive unsolicited help as undermining their competence (conveying to them that they might not be able to complete the task themselves) and autonomy (pressuring them to act in a certain way). Consequently, unsolicited help may lead children to perceive themselves as less competent, to like the task less, and to become less motivated.

Attribution theory (Graham 2020; Weiner 1972) postulates that children readily seek answers to why‐questions (i.e., attributions), such as “Why did my teacher help me?” Typically, these attributions differ in locus (internal, external), controllability (controllable, uncontrollable), and stability (stable, unstable). Unsolicited help can lead children to feel less competent, because children know that people are more likely to offer unsolicited help when they believe the recipient's struggles and failures stem from uncontrollable factors such as low ability (Graham 2020). Thus, when children receive unsolicited help, they may infer that others believe they have low ability. Because this is an attribution to an internal, uncontrollable, and stable cause, children may denigrate their ability and decide to withdraw from the task on hand (e.g., avoid challenges).

## Empirical Evidence

There is extensive literature on when and how parents and teachers provide help (e.g., Cooper et al. 2000; Ng et al. 2004; Park et al. 2016; Pomerantz et al. 2007; van de Pol et al. 2010; Vygotsky and Cole 1978) and when and how children seek help (Butler and Neuman 1995; Cluver et al. 2013; De Cooke and Brownell 1995; Nelson‐Le Gall 1987; Ryan et al. 2001; Selmeczy et al. 2023). A smaller literature has examined the psychological consequences of *receiving* help.

One line of experimental research suggests that children may infer that those who receive help are less competent. For example, U.S. children as young as 4 years thought that those who received help were less smart (Graham and Barker 1990; Sierksma and Shutts 2020). Another line of experimental research suggests that help can undermine motivation. For example, U.S. children ages 4–5 who received unsolicited help (“Hmm…this is hard, why don't I just do it for you”) persisted less on a subsequent task (Leonard et al. 2021). Similarly, U.S. children who received explicit instructions (e.g., telling them how to do a task) showed reduced exploration (Bonawitz et al. 2011; Yu et al. 2018). Cross sectional correlational studies in the United States found that children who received more intrusive help were less motivated (e.g., Cooper et al. 2000; Maloney et al. 2015; Pomerantz and Eaton 2000).

## Direct Versus Indirect Help

Not all help is alike, however. Adults provide different types of help (Bonawitz et al. 2011; Grolnick 2002; Klahr and Nigam 2004; Nadler and Chernyak‐Hai 2014; Pomerantz et al. 2007). When adults see a child struggling on a task, they might give the child the right answer (i.e., direct help, also known as dependency‐oriented or outcome‐oriented help) or teach the child strategies to find the right answer themselves (i.e., indirect help, also known as autonomy‐oriented or mastery‐oriented help). Adults may provide *direct help* when they believe the child is low in ability and may not be able to learn the skills needed to complete the task, regardless of how hard they try (Lee et al. 2022; Nadler and Chernyak‐Hai 2014). By giving the right answer, the adult helps the child complete the task without calling on the child's own ability. By contrast, adults may provide *indirect help* when they believe the child is high in ability and is able to learn the skills needed to complete the task (Bonawitz et al. 2011; Grolnick 2002; Ng et al. 2004). By giving a hint, the adult helps the child complete the task by calling on the child's own ability, giving them an opportunity to try again and learn. Thus, adults may provide indirect (vs. direct) help when they assume that the child's struggles do not stem from a lack of ability.

There is preliminary evidence that children, like adults (Nadler and Chernyak‐Hai 2014), pick up on the meaning of different types of help. Research in the Netherlands shows that, from age 7, children infer that those who receive direct help are less competent than those who receive indirect help (Sierksma 2023, Study 1), and children give more direct help than indirect help to those they perceive to be low in competence (Sierksma 2023, Study 2 and 3). These findings demonstrate that when children see another child receiving direct or indirect help, they draw different inferences about *the other child's* level of competence. Even if children perceive others who receive direct help as less competent, this does not mean that they would perceive *themselves* as less competent after receiving direct help, or that they would like the task less or be less motivated. The purpose of the present research was to investigate the psychological effects of receiving different types of help on children themselves.

## Contributions of the Present Research

Parents, teachers, and other professionals are often encouraged to offer children indirect help, as it is believed to be empowering. Several psychological theories suggest that indirect help is indeed more beneficial than direct help (Grolnick 2002; Nadler and Chernyak‐Hai 2014; Pomerantz and Eaton 2000), but causal evidence remains scarce. Our experimental research, for the first time, systematically examined the psychological effects of direct and indirect help on children's self‐perceptions, task perceptions, and motivation. Thus, our research has novel theoretical implications for the understanding of direct and indirect help.

One older experiment conducted in the United States (Shell and Eisenberg 1996) provides preliminary evidence, finding that girls (but not boys) felt less smart and more upset after receiving direct versus indirect help. However, this study lacked a no‐help control condition, making it impossible to determine whether help had beneficial or detrimental effects, and it did not examine the motivational consequences of help. Our work extends beyond this single experiment by systematically examining not only children's subjective experiences (e.g., self‐perceptions, liking of the help) but also their motivation (e.g., persistence, challenge seeking). These outcomes have real‐world importance: Children who feel competent, persevere, and embrace challenges tend to perform better in school (Blackwell et al. 2007; Burnette et al. 2013; Guay et al. 2004; Hecht et al. 2023; Morales et al. 2023; Sierksma and Shutts 2025; Spinath et al. 2006). Also extending previous work, our research employs a rigorous experimental design to establish causal effects of direct and indirect help relative to no help. By conducting three preregistered experiments, we ensure the robustness and replicability of our findings.

If results reveal that the effects of indirect help are similar to those of direct help, this would challenge conventional wisdom, making the theoretical and applied implications of our work even more significant. Given that psychological theory, parenting, and educational practice embrace indirect help as beneficial to children (Grolnick 2002; Pomerantz et al. 2007), knowing whether indirect help is beneficial or detrimental for development will advance our theoretical understanding of help and inform real‐world parenting and educational interventions on whether and how to offer help to children.

## Present Research

We conducted three preregistered experiments to examine the psychological consequences of receiving unsolicited help in middle childhood (ages 7–9). In each experiment, children worked on a series of puzzles and then received *direct help* (i.e., the right answer), *indirect help* (i.e., a hint), or *no help* from an experimenter. We examined how the help affected children's self‐perceptions (e.g., “I am not smart enough”), task perceptions (e.g., “This task is just too hard or no fun”), and motivation (e.g., challenge seeking and persistence). We integrated the results of our three experiments through an internal meta‐analysis (Study 4). Although not preregistered, this meta‐analysis allowed us to better understand the robustness of the findings across studies. All studies were conducted between 2020 and 2022 and approved by the Ethics Review Boards of the universities where the first author was employed (VCWE‐2020‐056, VCWE‐2021‐005, 22‐0263).

We focused on middle childhood (ages 7–9) for three reasons. First, children this age understand the difference between direct and indirect help (Sierksma 2023), making this a developmentally meaningful period to study the effects of direct and indirect help. Second, children this age often receive unsolicited help from parents and teachers (Cooper et al. 2000), making our research relevant for real‐world parenting and educational contexts. Third, at this age, self‐views are still relatively malleable (Brummelman and Thomaes 2017), highlighting potential for intervention.

We conducted the research in the Netherlands. Our total sample across the studies (*N* = 619, 7–9 years) was balanced in terms of gender (51% girls, 49% boys), representative of the Dutch population in terms of ethnicity (80% was native Dutch; CBS 2024), and mostly with above‐average socioeconomic status in terms of parental income and parental educational level (Table 1 presents all demographics across the three studies). By assessing these demographic characteristics, we were able to examine, in our internal meta‐analysis, whether effects were moderated by age, gender, ethnicity, parental income, and parental educational level. To protect children's privacy, we did not collect information on their place of residence.

**TABLE 1:** Overview of demographic data for each study.

	Study 1 (*N* = 251)	Study 2 (*N* = 194)	Study 3 (*N* = 174)
Mean age (SD)	8.1 (0.8)	7.9 (0.8)	8.0 (0.9)^a^
*Gender*			
% Boys	51.8	44.8	36.8
% Girls	46.6	49.5	45.4
% Did not report	0.8	5.7	17.8
% native Dutch^b^	79.7	83.0	78.2
% Did not report ethnicity		5.7	
*Gross annual household income* ^c^			
% < 35,000	9.6	12.9	6.9
% 35,000–70,500	29.9	34.0	20.1
% > 70,500	31.1	27.3	32.8
% Did not report	29.5	25.8	40.2
*Education parent 1 and 2* ^d^			
% High school or less	20.3–26.3	14.4–27.8	16.0–23.6
% Bachelor's or master's degree	66.1–72.9	60.3–79.9	54.0–63.2
% Did not report	6.8–7.6	5.7–11.9	20.7–22.4

*Note:* Demographics are in percentages of total sample, except for children's age.

^a^ Four parents did not report age.

^b^ In the Netherlands, about 80% of all youth (4–12 years) do not have a migration background (CBS 2024).

^c^ Median household income was € 36,000 in 2023 in the Netherlands (CBS 2024).

^d^ Educational levels are presented in ranges, because we assessed and analyzed them separately for two of the child's parents. For a complete list of ethnicities see Table S3.

We kept our experimental design consistent across studies. While each study used the same experimental manipulation—comparing the effects of direct help, indirect help, and no help—they were conducted in different contexts and used different dependent variables (for all differences, see Table S4). We adopted this approach because it allowed us to establish the replicability of our findings while at the same time exploring the generalizability of our findings across contexts and dependent variables. First, while Study 2 was conducted online in times of the COVID‐19 pandemic, Studies 1 and 3 were conducted in person outside of the pandemic. Second, unlike Study 1, Studies 2 and 3 indexed both *domain‐specific* self‐perceived ability (i.e., seeing oneself as capable at the task) and *general* self‐perceived ability (i.e., seeing oneself as capable in general). Third, unlike Study 1, Studies 2 and 3 examined the impact of help on children's persistence and challenge seeking. Self‐perceived ability, persistence, and challenge seeking are critical ingredients of academic success (Blackwell et al. 2007; Burnette et al. 2013; Hecht et al. 2023; Spinath et al. 2006). With these changes, we were able to establish whether the effects of help (a) occur in in‐person interactions, (b) affect children's self‐perceptions beyond the task on hand, and (c) have downstream consequences for motivation.

## 1 Study 1

The aim of Study 1 was to determine the causal effect of direct and indirect help on children's self‐perceptions, task perceptions, and motivation. We hypothesized that children who received direct help would report more negative self‐perceptions, more negative task perceptions, and lower task motivation than would children who received indirect help or no help.

Because Study 1 was our first study in this line of research, we included a broad set of items to measure self‐perceptions (i.e., self‐perceived ability, self‐perceived effort, self‐perceived potential for growth) and task perceptions (i.e., task liking, perceived task difficulty, perceived clarity of instructions). We also assessed children's task motivation (i.e., whether they wanted to do the task again). We assessed task liking because it is central to intrinsic motivation (Deci and Ryan 1985). We assessed perceived task difficulty and perceived clarity of instructions because children who find unsolicited help unpleasant might want to attribute it to external rather than internal sources (e.g., they may conclude that the instructions were unclear, so as to avoid the conclusion that they were not good at the task; Weiner 1972).

### 1.1 Method

#### 1.1.1 Participants

Data were collected in July and August 2020. Our preregistered target sample size was 64 children per condition (i.e., 192 children in total; based on a medium effect size, power = 0.80 and α = 0.05, two‐tailed; Cohen 1992). Because we suspected that some children would meet our preregistered exclusion criteria, we strived to recruit a minimum of 200 children. Data collection took place at NEMO Science Museum in Amsterdam (the Netherlands) for a set period of time (2 weeks). The museum draws families from across the country, not just from Amsterdam. We preregistered that we would include as many children as possible during this period, even if this number exceeded the desired number of 200. Given the lack of previous research on receiving help (and, consequently, on expected effect sizes), all recruited children were included in our analyses, maximizing statistical power. Because we did not analyze the data before the termination of data collection, the decision to terminate data collection could not have been influenced by the study results. Participants were 257 children (ages 7–9). As preregistered, children who expressed suspicion about the cover story (*n* = 6) were excluded. Our final sample consisted of 251 children. Table 1 presents details on age, gender, ethnicity, and socioeconomic status.

#### 1.1.2 Procedure

The experiment was programmed in Qualtrics and took about 15–20 min. Children sat in front of a laptop, wore headphones, and received instructions via prerecorded audio.

Children did a task (called the “block test”) consisting of several exercises (called “puzzles,” taken from Raven's Progressive Matrices, see OSF). Children first practiced with one puzzle. They were told that they would see puzzles with a piece missing: “You get to pick which piece is missing.” Several possible solutions were shown below each puzzle. Children then selected an answer and received performance feedback. If they picked the wrong answer (*n* = 17), they tried a second time. If picked the wrong answer a second time (*n* = 5), they received the correct answer. Subsequently, children were told: “You get to solve 7 puzzles for this test. You have to pick an answer for each puzzle.”

The first six puzzles were then presented to children in random order (see OSF for the puzzles). We piloted the puzzles with children aged 7–9 years and selected a set of puzzles that was moderately difficult and would yield a level of performance that would match the bogus feedback (i.e., they were told they solved 3 out of 6 puzzles). When they had finished six puzzles, children were told that the computer would check how many they answered correctly. Independent of their actual performance, the computer concluded that they had answered three correctly and three incorrectly. Children were then told: “Some children need help with the block test and others do not. Your answers will now be sent to the researcher. The researcher is going to think about whether you need help. When the researcher thinks you need help, they will also decide whether you need the correct answer or a hint.” Children thus received the help through the computer, but the help was framed as coming from an experimenter.

Children were shown a turning hourglass for 30 s after which they were told: “The researcher decided you need…” Children were randomly assigned to be informed that the researcher believed they needed “the correct answer,” “a hint,” or “no help.” In each condition, children were then shown the seventh puzzle. In the *direct help condition*, children were told the correct answer: “The correct answer is the one with the circle at the bottom.” In the *indirect help condition*, children received a hint: “Each shape occurs three times in the puzzle. For example, you see 3 squares. Perhaps you can find the right answer by finding out which shape does not occur three times in the puzzle.” In the *no help condition*, children were told the same as when they made the previous six puzzles: “You can now complete the last puzzle. Find the missing piece and then click on your answer.”

#### 1.1.3 Materials

Before the study, children practiced how to answer the questions by indicating how much they liked ice cream using a 6‐point smiley face scale. In the study, they completed the questions about self‐perceptions and task perception using the same scale (1 = *Not at all*, with a sad smiley, 6 = *A lot*, with a happy smiley). The constructs were presented in randomized order, and questions within each construct were presented in randomized order. Finally, children completed the task motivation measure and exploratory measures.

##### 1.1.3.1 Self‐Perceptions

Children reported their self‐perceived ability (i.e., “I am good at the block test”), self‐perceived effort (i.e., “I tried my best on the block test today”), and self‐perceived potential for growth (i.e., “I can always learn new things in the block test”). As preregistered, items were analyzed separately.

##### 1.1.3.2 Task Perception

Children reported their task liking (“I like the block test”), perceived task difficulty (“I think the block test is easy”), and clarity of instructions (“I think instructions for the block test were clear”).

##### 1.1.3.3 Task Motivation

Last, children were told: “Finally you can do another puzzle if you want to, you can decide for yourself! Do you want to do another puzzle?” We recorded whether children selected (vs. did not select) doing another puzzle.

##### 1.1.3.4 Exploratory Measures

Children reported (1) what type of help they received, if any (no help, a hint, the correct answer); and (2) how much help they thought they needed (1 = *Not at all*, 6 = *A lot*). In addition, they answered three open‐ended questions: (1) “Did you like receiving help [no help]?” (2) “Do you think something was fake or not real in the study?”, and (3) and “If there is something else you would like to say, you can do so here.” We do not report results for questions 1 and 3, because many children did not provide an answer or wrote unrelated comments (e.g., “Thank you for the study”). Question 2 probed suspicion for the cover story; as preregistered, we excluded children who indicated the feedback was not real or that they thought the experimenter did not really look at their answers. Afterwards, children were debriefed and thanked for participation, and they received a certificate for their participation.

#### 1.1.4 Data Analysis

We ran linear regression models on self‐perception and task‐attribution items (which are continuous variables). We ran a logistic regression on task motivation (which is a dichotomous variable). Because our hypotheses reflect comparisons between specific conditions (rather than an overall omnibus effect), we ran two linear regressions per dependent variable, including the experimental conditions as two dummy‐coded variables. We deviated from the preregistration by using dummy coding instead of contrast coding, because contrast coding cannot be performed if a condition variable has three levels. Model 1 always included direct help as the reference category (i.e., comparing indirect and no help to direct help). Model 2 always included no help as the reference category (i.e., comparing direct and indirect help to no help).

We used α = 0.05, two‐tailed. Although this was not preregistered, we corrected for multiple testing when we performed more than one test per hypothesis. This was the case for self‐perceptions (three tests per hypothesis) and task perceptions (two tests per hypothesis). Following Bonferroni correction, we divided α by the number of tests per hypothesis, leading to α = 0.017, two‐tailed, for self‐perceptions and α = 0.025, two‐tailed, for task perceptions. Note that for all studies we also explored whether the findings were moderated by children's age, gender, and performance on the task, and we report the results in the Supplementary File S1.

### 1.2 Results

Table 2 presents the results (means and standard deviations for all studies are reported in Tables S1 and S2). On average, and as intended, children solved 3.10 (out of 6) puzzles (SD = 1.24), and they perceived the task as moderately difficult (i.e., their difficulty rating was around the midpoint of the scale).

#### 1.2.1 Preregistered Confirmatory Analyses

See Table 2.

**TABLE 2:** Study 1 regression results.

Preregistered	*M* (*SD*)	Direct vs. no help (model 1)		Direct vs. indirect help (model 1)		Indirect vs. no help (model 2)	
		*β*	B 95% CI	*β*	B 95% CI	*β*	B 95% CI
Task liking	5.37 (0.91)	0.17*	0.32 [0.05, 0.59]	0.22**	0.43 [0.15, 0.70]	0.06	0.11 [−0.16, 0.38]
Task difficulty	3.88 (1.06)	0.17*	0.38 [0.06, 0.70]	0.06	0.15 [−0.18, 0.47]	−0.10	−0.23 [−0.55, 0.09]
Task instructions	5.14 (1.07)	0.11	0.26 [−0.7, 0.58]	0.07	0.16 [−0.17, 0.49]	−0.04	−0.10 [−0.43, 0.23]
Self‐ability	4.39 (1.00)	0.26***	0.59 [0.27, 0.91]	0.07	0.17 [−0.16, 0.49]	−0.18**	−0.42 [−0.75, 0.10]
Self‐effort	5.61 (0.74)	−0.02	−0.20 [−0.25, 0.20]	−0.06	−0.09 [−0.32, 0.13]	−0.04	−0.07 [−0.29, 0.16]
Self‐growth	4.94 (1.20)	−0.03	−0.07 [−0.43, 0.29]	−0.02	−0.05 [−0.42, 0.32]	0.01	0.02 [−0.35, 0.39]
Motivation	1.22 (0.42)	—	−0.43 [−1.26, 0.36]	—	−0.00 [−0.72, 0.72]	—	0.43 [−0.39, 1.22]
**Exploratory**							
Need	2.75 (1.23)	−0.38***	−1.00 [−1.35, −0.65]	−0.09	−0.23 [−0.58, 0.13]	0.29***	0.77 [0.42, 1.13]

*Note:* Model 1: direct help as reference category (i.e., comparing indirect and no help to direct help), model 2: no help as reference category (i.e., comparing direct and indirect help to no help).

* *p* ≤ 0.05.

** *p* ≤ 0.01.

*** *p* ≤ 0.001.

##### 1.2.1.1 Self‐Perceptions

Children who received *direct help* had lower self‐perceived ability than did children who received no help, *p* < 0.001. Children who received *indirect help* also had lower self‐perceived ability than did children who received no help, *p* = 0.011. Children who received *direct help* did not differ significantly from those who received *indirect help* in their self‐perceived ability, *p* = 0.311. By contrast, children's self‐perceived potential for growth and self‐perceived effort did not differ significantly between conditions, *ps* > 0.426.

##### 1.2.1.2 Task Perceptions

Children who received *direct help* liked the task less than did children who received no help, *p* = 0.021. Children who received *indirect help* did not differ significantly from those who received no help in how much they liked the task, *p* = 0.433. Children who received *direct help* also liked the task less than those who received *indirect help*, *p* = 0.002.

Furthermore, children who received *direct help* perceived the task as more difficult than did children who received no help, *p* = 0.021. Children who received *indirect help* did not differ significantly from those who received no help or direct help in how difficult they found the task, *p* = 0.161 and *p* = 0.374, respectively.

Children's perceived clarity of the task instructions did not differ significantly between conditions, *p* > 0.117.

##### 1.2.1.3 Task Motivation

Overall, 77.7% of children wanted to do another puzzle. Children's motivation did not differ significantly between conditions, *p*s > 0.262.

#### 1.2.2 Exploratory Analyses

##### 1.2.2.1 Perceived Need

Children who received *direct help* thought they needed more help than did children who received no help, *p* < 0.001. Children who received *indirect help* also thought they needed more help than did children who received no help, *p* < 0.001. Children who received *direct help* did not differ significantly from those who received *indirect help* in how much help they thought they needed, *p* = 0.210.

##### 1.2.2.2 Self‐Reported Type of Help Received

Afterwards, we asked children what kind of help they had received (i.e., no help, a hint, or the right answer): 22 children misreported the type of help they had received. A Chi‐square test comparing misreports across conditions was marginally significant, χ^2^(2) = 5.32, *p* = 0.070. Independent sample *t*‐tests show that children who received *direct help* were more likely to misreport (20.00%) than children who received no help (3.50%) or indirect help (2.50%), *t*(168) = 3.43, *p* < 0.001, and *t*(164) = 3.67, *p* < 0.001, respectively.

### 1.3 Discussion

In Study 1, receiving unsolicited help undermined children's self‐perceived ability and caused children to believe they needed more help, regardless of whether the help was direct or indirect. Unlike receiving indirect help, receiving direct help also led children to like the task less and to perceive it as more difficult. However, receiving unsolicited help did not impact children's task motivation. Interestingly, a substantial proportion of children (8.80%) misreported the type of help that they received, especially direct help.

## 2 Study 2

The aim of Study 2 was to replicate the Study 1 findings and to improve our measure of task motivation. We included both a challenge seeking measure in which children could choose between easy and difficult puzzles (Brummelman et al. 2014) and a persistence measure that indexed how long children spent trying to solve an unsolvable puzzle (Leonard et al. 2021).

We hypothesized that children who receive direct help would report more negative self‐perceptions, more negative task perceptions, and lower task motivation than children who receive indirect help or no help. In addition, we explored whether children who receive direct help would be more likely to misreport the type of help they received than would children who receive indirect help or no help. We also explored whether children who receive direct help would think they needed more help and would dislike the help more than children who receive no help.

### 2.1 Method

#### 2.1.1 Participants

Data were collected between March and November 2021. Study 1 showed a medium effect size of direct help versus no help on self‐perceived ability (Cohen's *d* = 0.54). An a priori power analysis showed that we needed 64 children per condition (i.e., 192 children in total) to identify a medium effect size (with power = 0.80 and α = 0.05, two‐tailed). We preregistered that we would stop collecting data once we had a total of 192 eligible participants (i.e., excluding children who met our preregistered exclusion criteria).

Children were recruited via social media ads and lived in the Netherlands. Participants were 209 7‐to‐9‐year‐old children. As preregistered, 15 children were excluded: 11 expressed suspicion, 1 child participated twice (we excluded the second observation), and 3 participants experienced interference during the session. Our final sample size consisted of 194 children. Thus, we included two more children than we had preregistered because our experimenters continued data collection without realizing we had met our preregistered sample size. We include the full sample in our analyses because we did not analyze the data before termination of data collection, so the decision to terminate data collection could not have been influenced by the study results. Table 1 presents details on age, gender, ethnicity, and socioeconomic status.

#### 2.1.2 Procedure and Materials

Due to the COVID‐19 pandemic, we tested children online via live Zoom sessions. Children completed the study via a Qualtrics survey while an experimenter provided instructions. Children wore a headphone and were visible (both to themselves and to their experimenter) via their webcam while completing the study. The experimenter turned off their own camera, so as to minimize children's feeling of being observed, but they told children they were present in case they had any questions. The experimental design was identical to Study 1, but we did revise some dependent variables and added some dependent variables.

#### 2.1.3 Measures

The order of self‐ and task perceptions was counterbalanced, and questions within each construct were randomized.

##### 2.1.3.1 Self‐Perceptions

While Study 1 measured only task‐specific self‐perceived ability, Study 2 measured both task‐specific and general self‐perceived ability. While Study 1 included one item to measure task‐specific self‐perceived ability, Study 2 included three: “I think I am good at the block test” (as in Study 1), “I think I can do well at the block test,” and “I think I have a talent for the block test” (Cronbach's α = 0.80). We also measured children's general self‐perceived ability using three items: “I feel smart,” “I think I am a smart person,” and “I am someone who can learn well” (Cronbach's α = 0.76). As preregistered, because the Cronbach's α was high for each scale (> 0.70), we averaged across items for each scale separately.

##### 2.1.3.2 Task Perceptions

As in Study 1, children reported task liking (“I like the block test”) and task difficulty (“I think the block test is easy”). Deviating from Study 1, we did not measure perceived clarity of task instructions, because Study 1 revealed no condition effects on it.

##### 2.1.3.3 Task Motivation

We measured children's challenge seeking and persistence. To index *challenge seeking*, we told children that they were going to solve another set of four puzzles but that they could choose which ones. For each set, children could choose between easy and difficult puzzles (based on Brummelman et al. 2014; Mueller and Dweck 1998). Children were told: “If you choose to this block test, you might make many mistakes, but you'll definitely learn a lot too. But if you choose this block test, you won't make many mistakes, but you won't learn much either.” Challenge seeking was computed as the number of difficult puzzles selected.

To index *persistence*, we told children there was one more puzzle that they had to solve. We told children the puzzle was planned beforehand so that children would not think the persistence puzzle was selected based on their answers to the challenge seeking measure. Children were then given a puzzle that, unbeknownst to them, was unsolvable (see OSF). Children could quit the task at any time by pressing a button. Persistence was computed as the number of seconds children spent trying to solve the puzzle before quitting. As preregistered, we excluded 7 outliers on this measure (using the outlier‐labeling rule; Hoaglin and Iglewicz 1986).

##### 2.1.3.4 Exploratory Measures

As in Study 1, children reported (1) what type of help they received, if any (no help, a hint, the correct answer); (2) how much help they thought they needed; and (3) provided an open‐ended response to: “Do you think something was fake or not real in the study?” We added two new questions. First, we asked children: “Did you like receiving no help [a hint, the correct answer] on the last block test?” (1 = *Not at all*, 6 = *A lot*). This latter question was routed based on what type of help children *reported to have received* (not what they actually received), so we had to exclude children who misreported the type of help they had received from the analyses of this variable. Second, we asked children how many puzzles they thought they solved (0–6). Afterwards, children were debriefed and thanked.

#### 2.1.4 Data Analysis

As in Study 1, for our preregistered analyses, we performed linear regression analyses for each dependent variable, using dummy variables to index the three experimental conditions. We used α = 0.05, two‐tailed. As in Study 1, although this was not preregistered, we corrected for multiple testing (i.e., we used α = 0.025, two‐tailed, for task perception and task motivation).

### 2.2 Results

Table 3 presents the results. On average, and as intended, children solved 3.60 (out of 6) puzzles (SD = 1.29), and they perceived the task as moderately difficult.

**TABLE 3:** Study 2 regression results.

Preregistered	*M* (SD)	Direct vs. no help (model 1)		Direct vs. indirect help (model 1)		Indirect vs., no help (model 2)	
		*β*	B 95% CI	*β*	B 95% CI	*β*	B 95% CI
Task liking	5.30 (1.00)	−0.00	−0.00 [−0.36, 0.35]	−0.08	−0.18 [−0.53, 0.18]	−0.08	−0.17 [−0.51, 0.17]
Task difficulty	3.77 (1.16)	−0.05	−0.12 [−0.53, 0.29]	−0.04	−0.10 [−0.51, 31]	0.01	0.02 [−0.37, 0.42]
Ability task	4.21 (0.98)	−0.03	−0.05 [−0.40, 0.30]	−0.08	−0.17 [−0.52, 0.17]	−0.06	−0.12 [−0.46, 0.22]
Challenge seeking	2.02 (1.26)	0.03	0.07 [−0.39, 0.51]	0.05	0.12 [−0.32, 0.57]	0.02	−0.06 [−0.37, 0.49]
Persistence	56.06 (21.4)	0.02	0.91 [−6.73, 8.54]	0.10	4.61 [−3.11, 12.33]	0.08	3.71 [3.80, 11.21]
**Exploratory**							
Ability general	5.12 (0.88)	−0.00	−0.00 [−0.31, 0.31]	−0.11	−0.19 [−0.50, 0.12]	−0.10	−0.19 [−0.49, 0.11]
Like receiving help	4.48 (1.47)	0.26**	0.79 [0.21, 1.37]	0.29**	0.89 [0.30, 1.48]	0.03	0.10 [−0.42, 0.62]
Need	2.74 (1.30)	−0.16	−0.43 [−0.89, 0.03]	−0.01	−0.02 [−0.47, 44]	0.15	0.41 [−0.03, 0.85]

*Note:* Model 1: direct help as reference category (i.e., comparing indirect and no help to direct help), model 2: no help as reference category (i.e., comparing direct and indirect help to no help).

** *p* ≤ 0.01.

#### 2.2.1 Preregistered Confirmatory Analyses

Children's task‐specific self‐perceived ability (*p*s > 0.328), general self‐perceived ability (*p*s > 0.211), task liking (*p*s > 0.320), perceived task difficulty (*p*s > 0.554), challenge seeking (*p*s > 0.583), persistence (*p*s > 0.211), and perceived need for help (*p*s > 0.068) did not differ significantly between conditions.

Children liked receiving *direct help* less than receiving *no help*, *p* = 0.008. Children did not like receiving *indirect help* significantly less or more than receiving no help, *p* = 0.699. Children liked receiving *direct help* less than receiving *indirect help*, *p* = 0.003.

#### 2.2.2 Exploratory Analyses

##### 2.2.2.1 Self‐Reported Type of Help Received

A Chi‐square test comparing misreports across conditions was significant, χ^2^(2) = 12.41, *p* = 0.002. Independent sample *t*‐tests show that children who received *direct help* were more likely to misreport (33.3%) than children who received no help (4.5%) or indirect help (14.0%), *t*(124) = 4.47, *p* < 0.001, and *t*(125) = 2.48, *p* = 0.015, respectively.

##### 2.2.2.2 Perceived Performance

On average, children believed they had solved 4.96 puzzles (SD = 1.33). Children's self‐perceived performance did not differ significantly between conditions, *p*s > 0.090.

### 2.3 Discussion

Study 2 sought to replicate our Study 1 findings. Study 2 did not replicate the effects of help on children's self‐perceptions or task perceptions. It did, however, replicate the effects of help on misreporting: Children who received direct help were more likely than those who received indirect help or no help to misreport the type of help they had received. Whereas Study 1 was conducted in person before the COVID‐19 pandemic, Study 2 was conducted via Zoom during the COVID‐19 pandemic. To establish the robustness of our Study 1 findings, and to examine whether the non‐replication of Study 2 was potentially due to its context and timing, we conducted a third and final study. Like Study 1, Study 3 was conducted in person.

## 3 Study 3

The aim of Study 3 was to replicate the Study 1 findings, using the improved dependent variables of Study 2. We hypothesized that children who received direct help (vs. indirect help) would report (1) more negative task‐specific self‐perceptions, (2) more negative task attributions, (3) lower task motivation, (4) be more likely to misreport the type of help they received, (5) indicate needing more help, and (6) dislike receiving direct help more.

### 3.1 Method

#### 3.1.1 Participants

Data were collected in July 2022. An a priori power analysis showed that we needed 56 children per condition (i.e., 168 children in total) to identify a medium effect size (Cohen's *d* = 0.54) with power = 0.80 and α = 0.05, two‐tailed. Like in Study 1, data collection took place at NEMO Science Museum in Amsterdam (the Netherlands) for a set period of time (2 weeks). Similar to Study 1, we preregistered that we would recruit as many children as possible during this period and include them all in our analyses, maximizing statistical power. Because we did not analyze the data before the termination of data collection, the decision to terminate data collection could not have been influenced by the study results. Participants were 190 children (7‐to‐9 years). As preregistered, 16 children were excluded (3 expressed suspicion about the performance feedback and 13 were outside the age range). Table 1 presents details on age, gender, ethnicity, and socioeconomic status.

#### 3.1.2 Procedure, Materials, and Data Analysis

We conducted Study 3 in the same location as Study 1, using the same experimental design and materials as Study 2, with a minor difference: Children also reported their general self‐perceived ability before taking the task, so that we could control for potential baseline differences. The multiple‐item scales were internally consistent: pre‐task general self‐perceived ability (Cronbach's α = 0.75), post‐task general self‐perceived ability (Cronbach's α = 0.86), and post‐task task‐specific self‐perceived ability (Cronbach's α = 0.81). We used α = 0.05, two‐tailed. As in Studies 1 and 2, although this was not preregistered, we corrected for multiple testing (i.e., we used α = 0.025, two‐tailed, for task perception and task motivation).

### 3.2 Results

Table 4 presents the results. On average, and as intended, children solved 3.69 (out of 6) puzzles (SD = 1.09), and they perceived the task as moderately difficult.

**TABLE 4:** Study 3 regression results.

	*M* (SD)	Direct vs. no help (model 1)		Direct vs. indirect help (model 1)		Indirect vs., no help (model 2)	
		*β*	B 95% CI	*β*	B 95% CI	*β*	B 95% CI
**Preregistered**							
Task liking	5.39 (0.89)	0.21*	0.39 [0.07, 0.72]	0.04	0.08 [−0.25, 0.40]	−0.17	0.32 [−0.64, 0.01]
Task difficulty	4.00 (1.11)	0.05	0.12 [−0.29, 0.53]	−0.03	−0.07 [−0.47, 0.34]	−0.08	−0.19 [−0.59, 0.22]
Ability task	4.30 (0.96)	0.14	0.28 [−0.07, 0.64]	0.03	0.06 [−0.30, 0.41]	−0.11	−0.23 [−0.58, 0.12]
Challenge seeking	1.82, (1.21)	0.31***	0.78 [0.36, 1.21]	0.02	0.05 [−0.38, 0.47]	−0.29***	−0.73 [−1.16, −0.31]
Persistence	58.53 (26.81)	−0.09	−7.14 [−7.02, 21.30]	−0.05	−4.00 [−18.05, 10.04]	−0.14	−11.14 [−25.19, 2.90]
Like receiving help	4.78 (1.34)	0.26**	0.72 [−0.17, 1.26]	0.27**	0.75 [0.20, 1.31]	0.02	0.04 [−0.49, 0.57]
Need	2.73 (1.28)	−0.35***	−0.95 [−1.39, −0.50]	0.02	0.05 [−0.39, 0.49]	0.37***	1.00 [0.55, 1.44]
**Exploratory**							
Ability general	5.02 (0.95)	0.12	0.23 [−0.12, 0.58]	0.05	0.10 [−0.25, 0.45]	−0.07	−0.13 [−0.48, 0.21]

*Note:* Model 1: direct help as reference category (i.e., comparing indirect and no help to direct help), model 2: no help as reference category (i.e., comparing direct and indirect help to no help).

* *p* ≤ 0.05.

** *p* ≤ 0.01.

*** *p* ≤ 0.001.

#### 3.2.1 Preregistered Confirmatory Analyses

##### 3.2.1.1 Self‐Perceptions

Children's general or task‐specific self‐perceived ability did not differ significantly between conditions, *p*s > 0.191.

##### 3.2.1.2 Task Perceptions

Children who received *direct help* liked the task less than did children who received no help, *p* = 0.018. Children who received *indirect help* did not differ significantly from those who received no help in how much they liked the task, *p* = 0.054. Children who received *direct help* did not differ significantly from those who received *indirect help* in how much they liked the task, *p* = 0.638. Children's perceived task difficulty did not differ significantly between conditions, *p*s > 0.361.

##### 3.2.1.3 Task Motivation

Children who *directed help* sought fewer challenges than did children who received no help, *p* < 0.001. Children who received *indirect* help sought fewer challenges than did children who received no help, *p* < 0.001. Children who received direct help did not differ significantly from those who received indirect help in how much help they sought, *p* = 0.819.

We identified one outlier on persistence; this participant spent 920 s, whereas all other children spent between 12 and 313 s. Regardless of whether the outlier was excluded, children's persistence did not differ significantly between conditions, *p*s > 0.065.

##### 3.2.1.4 Self‐Reported Type of Help Received

A Chi‐square test comparing misreports across conditions was significant, χ^2^(2) = 8.37, *p* = 0.015. Independent sample *t*‐tests show that children who received *direct help* were more likely to misreport (28.1%) than children who received no help (8.9%), *t*(111) = 2.67, *p* = 0.009, but they were not significantly more or less likely to misreport than children who received indirect help (20.7%), *t*(113) = 0.92, *p* = 0.361.

##### 3.2.1.5 Perceived Need

Children who received *direct help* thought they needed more help than did children who received no help, *p* < 0.001. Children who received *indirect help* thought they needed more help than did children who received no help, *p* < 0.001. Children who received *direct help* did not differ significantly from those who received *indirect help* in how much help they thought they needed, *p* = 0.819.

##### 3.2.1.6 Liking

As in Study 2, we had to exclude children who misreported the type of help they received. Children liked receiving *direct help* less than receiving no help, *p* = 0.011. Children did not like *indirect help* less or more than receiving no help, *p* = 0.877. Children liked receiving *direct help* less than receiving *indirect help*, *p* = 0.008.

#### 3.2.2 Exploratory Analyses

##### 3.2.2.1 Pre‐Task General Self‐Perceived Ability

We conducted two sets of non‐preregistered analyses. First, we repeated the analyses for post‐task general self‐perceived ability, controlling for pre‐task general self‐perceived ability. Children who received *direct help* had lower general self‐perceived ability than did children who received no help, *B* = 0.26, SE = 0.12, *p* = 0.029, 95% CI [0.03, 0.49]. Children who received *indirect help* did not differ significantly from those who received no help in their general self‐perceived ability, *B* = −0.02, SE = 0.12, *p* = 0.890, 95% CI [−0.24, 0.21]. Children who received *direct help* had lower general self‐perceived ability than did children who received *indirect help*, *B* = 0.24, SE = 0.12, *p* = 0.041, 95% CI [0.01, 0.47].

Second, we repeated the analyses for post‐task‐specific self‐perceived ability, controlling for pre‐task general self‐perceived ability. Children who received *direct help* did not differ significantly from those who received no help or *indirect help* in their task‐specific self‐perceived ability, *B* = 0.30, SE = 0.16, *p* = 0.067, 95% CI [−0.02, 0.62], and *B* = −0.14, SE = 0.16, *p* = 0.398, 95% CI [−0.46, 0.18], respectively. Children who received *indirect help* did not differ significantly from those who received no help in their task‐specific self‐perceived ability, *B* = −0.16, SE = 0.16, *p* = 0.318, 95% CI [−0.48, 0.16].

##### 3.2.2.2 Perceived Performance

On average, children believed they had solved 3.67 puzzles (SD = 1.23). Children's self‐perceived performance did not differ significantly between conditions, *p*s > 0.228.

### 3.3 Discussion

Study 3 largely replicates the Study 1 findings. This suggests that the non‐replication in Study 2 can be attributed to the context and timing of the study: online, without face‐to‐face interaction, in the midst of a COVID‐19 pandemic. Study 3 reveals that children who received help had lower general self‐perceived ability (in particular after controlling for baseline general self‐perceived ability) liked the task less and thought they needed more help, but they did not have lower task‐specific self‐perceived ability and did not perceive the task as more difficult. Also, children who received help sought fewer challenges, regardless of whether the help was direct or indirect. Compared to children who received no help or indirect help, those who received direct help were more likely to misreport the type of help they received and to dislike the help they received.

Receiving help reduced children's challenge seeking but not their persistence. If the experimenter had been there in person, children could have inferred that the experimenter who had offered help would provide hands‐on help on the persistence task, leading them to persist less. However, the experimenter was not there in person, which could explain why we did not find an effect. This suggests that children's lowered self‐perceived ability after help does not necessarily lead them to persist less, calling into question the idea that self‐perceived ability mediates the relationship between unsolicited help and persistence.

## 4 Study 4: Internal Meta‐Analysis

Because our results differed between studies, we conducted an internal meta‐analysis by aggregating data across studies. We focused on the measures that were included in all three studies: task‐specific self‐perceived ability (“I am good at the block test”), task liking, perceived task difficulty, perceived need for help, and misreporting. Results are reported in Table 5 (see supplementary file S1 for means and standard errors, Figure S1).

**TABLE 5:** Internal meta‐analysis regression results.

	Direct vs. no help (model 1)		Direct vs. indirect help (model 1)		Indirect vs. no help (model 2)	
	*β*	B 95% CI	*β*	B 95% CI	*β*	B 95% CI
Task liking	0.12**	0.24 [0.06, 0.42]	0.07	0.14 [−0.04, 0.32]	−0.50	−0.10 [−0.28, 0.08]
Task difficulty	0.06	0.15 [−0.07, 0.36]	0.00	0.01 [−0.21, 0.22]	−0.06	−0.14 [−0.35, 0.07]
Ability task	0.14***	0.32 [0.12, 0.53]	0.01	0.03 [−0.17, 0.24]	−0.13***	−0.29 [−0.49, −0.09]
Need	−0.30***	−0.81 [−1.04, −0.57]	−0.03	−0.09 [−0.33, 0.15]	0.27***	0.72 [0.49, 0.96]

*Note:* Model 1: direct help as reference category (i.e., comparing indirect and no help to direct help), model 2: no help as reference category (i.e., comparing direct and indirect help to no help).

** *p* ≤ 0.01.

*** *p* ≤ 0.001.

### 4.1 Self‐Perceived Ability

Children who received *direct help* or *indirect help* had lower self‐perceived ability than did children who received no help, *ps* < 0.001. Children who received direct help did not differ significantly from those who received indirect help in their self‐perceived ability, *p* = 0.757.

### 4.2 Task Liking

Children who received *direct help* liked the task less than did children who received no help, *p* < 0.009. Children who received *indirect help* did not differ significantly from those who received *direct help* or no help in how much they liked the task, *p* = 0.133 and *p* = 0.255, respectively.

### 4.3 Perceived Task Difficulty

Children's perceived task difficulty did not differ significantly between conditions, *p* > 0.172.

### 4.4 Perceived Need

Children who received *direct help* or *indirect help* thought they needed more help than did children who received no help, *ps* < 0.001. Children who received direct help did not differ significantly from those who received indirect help in how much help they thought they needed, *p* = 0.463.

### 4.5 Self‐Reported Type of Help Received

A Chi‐square test comparing misreports across conditions was significant, χ^2^(2) = 28.26, *p* < 0.001 (see Table 6). Independent sample *t*‐tests show that children who received *direct help* were more likely to misreport (26.24%) than did children who received no help (5.31%) or indirect help (11.65%), *t*(407) = 6.07, *p* < 0.001, and *t*(406) = 3.82, *p* < 0.001. Of all children who misreported the type of help received, 60.23% were in the direct help condition, compared to 27.27% and 12.50% in the indirect‐ and no‐help conditions (direct vs. indirect help: *t*(75) = 2.71, *p* = 0.008; direct help vs. no help: *t*(62) = −4.48, *p* < 0.001). Children who received indirect help rarely reported that they received direct help; only one of them did.

**TABLE 6:** Type of help received versus reported across all studies.

Type of help received	Type of help reported			
	Direct help	Indirect help	No help	Total misreports
Direct help	149	21	32	53
Indirect help	1	182	23	24
No help	1	10	196	11

None of the significant main effects of help reported were moderated by children's age, *p*s > 0.454, gender, *ps* > 0.057, or task performance, *ps* > 0.102. We explored how ethnicity (Dutch vs. non‐Dutch), household income, and parental educational level moderated the impact of help (see Data S1). Only the interaction involving parental educational level was significant: Children who received direct help (vs. no help) perceived the task to be more difficult, especially if their parents had higher educational levels.

## 5 General Discussion

Receiving help is a common experience for children (Cooper et al. 2000). In many cases, receiving help benefits children's academic and social development (Ryan et al. 2001; Zimmerman 2002). Often, however, children receive unsolicited help, which could have unintended consequences. Here, we present three preregistered studies on the psychological consequences of receiving unsolicited direct and indirect help. Our internal meta‐analysis, combining results from the three experiments, shows that children who received help (vs. no help) felt less competent, liked the task less, felt more in need of help, and often denied having received help—regardless of whether the help was direct or indirect. Receiving help, whether direct or indirect, also reduced children's challenge seeking in Study 3. Overall, there were few differences between direct and indirect help, except that children who received direct help disliked and misreported it more. Thus, despite being well‐intentioned, both direct and indirect help can be aversive and can lead children to denigrate their ability and experience a drop in motivation. These results have important implications for our understanding of how well‐intentioned and seemingly benign actions can backfire.

### 5.1 Theoretical Implications

Our findings are consistent with psychological theories predicting that unsolicited help can have adverse consequences. Self‐determination theory (Ryan and Deci 2000) suggests that receiving unsolicited help could thwart the needs for competence and autonomy, which could in turn undermine children's intrinsic motivation. Attribution theory (e.g., Graham 2020; Weiner 1972) suggests that receiving unsolicited help could undermine self‐perceived ability. Our results are consistent with these perspectives, showing that unsolicited help leads children to feel less competent, to think they need more help, and to avoid challenging tasks. Extending these theories, our work shows that children found receiving help aversive. They reported disliking it, and they misreported the type of help they had received—especially direct help. Direct help may feel more aversive because, unlike indirect help, it deprives children of the opportunity to practice their skills and accomplish the task themselves, thus undermining their autonomy.

Our work also extends existing research on direct and indirect help. Scholars (as well as parents, teachers, and other professionals) tend to assume indirect help is empowering (Burhan and van Leeuwen 2016; Grolnick 2002; Nadler and Chernyak‐Hai 2014; Pomerantz et al. 2007), signaling to children that they have the ability to complete a task themselves. Our results challenge this assumption: Although indirect help felt less aversive than direct help, it had largely similar effects on children's self‐perceived ability, perceived task difficulty, and perceived need of help. Why? This could be explained by the developmental focus of our work: ages 7–9. Research in the United States shows that, by age 5, children infer that students are less competent if they need to exert greater effort to complete a task (Muradoglu and Cimpian 2020) or receive help (Graham and Barker 1990; Sierksma and Shutts 2020). Yet, this inference might become more pronounced from age 10–12 (Graham and Chen 2020), especially after the transition to secondary school. This is when school environments become more formal, evaluative, and competitive, valuing children's performance over their growth or effort (Amemiya and Wang 2018; Eccles et al. 1993; see Cimpian 2017). Such environments may cultivate the belief that high‐ability students are those who learn effortlessly, without needing any direct help. Consequently, in secondary school, direct help may be a stronger low‐ability cue than indirect help. We call for research with a wide age range, from early childhood to adolescence, to examine developmental change in children's responses to direct and indirect help.

There were two domains in which direct and indirect help did lead to different responses: Children disliked receiving direct help more, and children who received direct help were more likely to misreport the type of help they received. One possibility is that children wanted to “hide” that they received direct help. This could reflect an underlying feeling of shame (Tangney and Dearing 2003), although our work did not directly test this mechanism. Overall, it seems that direct help elicited stronger negative *affective* reactions (e.g., dislike) than did indirect help, but that the two types of help solicited similar *cognitive* reactions (e.g., low self‐perceived ability). Perhaps direct and indirect help differ so subtly that it takes repeated exposure for young children to draw different inferences about themselves (e.g., “I'm not smart enough”) or the task (e.g., “I don't like this task”).

Although seemingly benign, unsolicited help can have adverse downstream consequences. Study 3 shows that, after receiving help, children were less likely to embrace challenges: they preferred easy tasks (which minimized the risk of failure but involved no opportunity for learning) over difficult tasks (which entailed the risk of failure but maximized the opportunity for learning). It is particularly noteworthy that this was the case for both direct and indirect help, with educators often providing indirect help in an attempt to motivate students (e.g., van de Pol et al. 2010). Embracing challenges is critical for children's development. Children who take on more challenges tend to perform better academically, and interventions that raise challenge seeking tend to improve academic achievement, especially for students who lag behind or belong to intellectually stigmatized groups (Blackwell et al. 2007; Burnette et al. 2013; Hecht et al. 2023). Thus, by reducing challenge seeking, unsolicited help—whether direct or indirect—could lead children to miss out on critical learning experiences.

Our findings provide novel insight into the development of self‐perceived ability. Children construct their self‐perceived ability based on their successes and failures, their social comparisons, and the messages they receive from parents and teachers (Brummelman and Thomaes 2017; Haimovitz and Dweck 2016; Wigfield et al. 2015). Contributing to this work, we show that children also learn about their abilities through the help that they receive—independent of their actual achievements. Our results show that receiving unsolicited help, whether direct or indirect, can lead children to develop lower self‐perceived ability. This concurs with earlier work in the United States showing that children tend to infer that those who receive help are less competent than those who do not (Graham and Barker 1990; Sierksma and Shutts 2020), and one study suggesting that children might feel threatened after receiving help (Shell and Eisenberg 1996). Interestingly, in Study 1, receiving unsolicited help undermined children's self‐perceived ability, but it did not undermine their self‐perceived effort or potential for growth. This suggests that unsolicited help conveys to children that they are currently not competent enough to solve the task on hand—not that they did not work hard enough, or that they would not be able to cultivate their competence in the future.

Although our internal meta‐analysis showed a consistent pattern of findings, the results differed between the three preregistered experiments. Unlike Study 1 and Study 3, Study 2 showed few significant effects of help. What explains the non‐replication in Study 2? We used similar designs across studies, but context and timing differed. Study 1 and Study 3 were conducted in person at a science museum, whereas Study 2 was conducted remotely via Zoom during the COVID‐19 pandemic. This could have affected the impact of help in two ways. First, in Study 2, children might have been distracted (because they were in lockdown with their family members), disengaged (because there was no real‐life face‐to‐face interaction with an experimenter), or mentally occupied (because children showed increased worries during the pandemic; e.g., Rothe et al. 2021). Second, in Study 2, children were observed by the experimenter and could observe themselves via their webcams. This might have increased their self‐awareness and self‐presentational concerns, leading them to think more about themselves (Geller and Shaver 1976) and to present themselves more favorably (Baumeister and Hutton 1987). Consequently, they may have been more reluctant to report negative inferences about themselves or the task.

### 5.2 Strengths, Limitations, and Future Directions

Our research has several strengths, including its experimental design, internal replications, and novel focus on the consequences of direct and indirect help for children's self‐perceptions, task perceptions, and motivation. Our research also has limitations. First, we focused on two specific forms of direct and indirect help. We provided children with the right answer (direct help) or a hint (indirect help). Although representative of many forms of direct and indirect help (Nadler and Chernyak‐Hai 2014; Pomerantz et al. 2007), we encourage researchers to diversify the types of help they study (e.g., providing children with an instruction sheet containing hints, which they can consult when needed, so that they do not have to overcome the barriers to asking another person for help).

Second, we conducted the three studies in the Netherlands, an individualistic country where children are socialized to be independent, autonomous, and self‐reliant individuals (Greenfield et al. 2003). Receiving or asking for help, then, might conflict with this core cultural value. Our findings may not necessarily generalize to collectivistic countries. We encourage researchers to explore potential cross‐cultural differences in the solicitation and receipt of help.

Third, we conducted our experiments in controlled settings. We encourage researchers to conduct field experiments examining the effects of help provided by key figures (e.g., teachers) on the development of children's self‐views, motivation, and achievement (e.g., grades, test scores).

Our findings generate important directions for future work. First, we call for research on the boundary conditions of the effects of help. Our work focuses specifically on unsolicited help provided by an adult authority figure in a one‐on‐one interaction. Would help be less threatening to children when they solicit it themselves? Would help be less threatening when provided by a peer, whose opinions might be less consequential for children's self‐perceived ability? And would help be less threatening when provided to the whole classroom, rather than to one child in particular? If so, why would help be less threatening in these cases (e.g., because it is less likely to trigger low‐ability attributions or shame)? These questions can be addressed through randomized experiments in field settings, bridging the gap between the lab and the field. Such research can reveal the importance of the *nature* (e.g., solicited vs. unsolicited), *source* (e.g., teacher vs. classmate), *context* (e.g., one‐on‐one vs. classroom‐based), and *mechanisms* (e.g., attributions, shame) of help.

Second, future studies should also consider including a wider age range to deepen our understanding of children's emotional responses to help. Our research focuses on ages 7–9, so our results may not necessarily generalize to other age groups. Self‐conscious emotions such as shame emerge early in life (Lewis and Sullivan 2013; Nikolić et al. 2023). We speculate that children find receiving direct help shameful, but we did not directly measure shame. We call for research that indexes shame through coding facial expressions and bodily postures after help, which would enable researchers to study shame in young children who are not able to provide reliable self‐reports.

Third, our findings provide insight into inequality in the classroom. Adults and children provide more help—especially direct help—to individuals who belong to intellectually stigmatized groups (Burhan and van Leeuwen 2016; Nadler and Chernyak‐Hai 2014; Sierksma et al. 2018), perhaps because they believe these individuals have lower ability (Durante et al. 2017; Fiske et al. 2002). Unsolicited help could thus perpetuate group‐based biases. For example, teachers may, unknowingly and unintentionally, offer more unsolicited help to children who belong to intellectually stigmatized groups, such as students from low socioeconomic or immigrant backgrounds (Brummelman and Sedikides 2023; Schoneveld and Brummelman 2023; Sierksma 2023). If these children have pre‐existing negative self‐views (Brummelman and Sedikides 2023), they may also be more likely to draw negative inferences from the help. Thus, by providing help unequally, teachers may inadvertently perpetuate existing inequalities (e.g., by lowering these students' self‐perceived ability). There is some supportive evidence in U.S. adults, with students from stigmatized groups experiencing lower self‐esteem and more depressed affect after receiving unsolicited help from a non‐stigmatized other (Schneider et al. 1996). In our internal meta‐analysis, most effects of help did not differ depending on children's ethnicity or socioeconomic status (i.e., household income or parental educational level). Our samples were fairly representative of the general Dutch population of children (CBS 2024), meaning that only a minority of children had non‐Western migration backgrounds or were from low socioeconomic status families. To address the generalizability of our findings, we encourage researchers to intentionally recruit more diverse samples.

### 5.3 Conclusion

In many cases, providing children with help is undoubtedly beneficial to their learning. Our research shows, however, that providing children with unsolicited help may inadvertently undermine children's self‐perceived ability, liking of the task, and willingness to embrace challenges—even if the help is provided in indirect ways, like offering a hint. This may have negative downstream consequences because children who have low self‐perceived ability, dislike school activities, avoid challenges, and tend to perform more poorly in school (Guay et al. 2004). Our work provides an important basis for research to examine how to provide help more wisely so as to benefit the learning of all children.

## Conflicts of Interest

The authors declare no conflicts of interest.

## Supporting information


Data S1.

## Data Availability

For each study, we preregistered our study design, hypotheses, and data analysis plans via OSF (Study 1: https://osf.io/mjkrg, Study 2: https://osf.io/v3m62, Study 3: https://osf.io/9ndh7) and we made our materials, data, and code available via OSF at: https://osf.io/t9mep.
