# Supplementary material for: “Here, Let Me Do It for You”: Psychological Consequences of Receiving Direct and Indirect Help in Childhood
Source: Child Dev. 2025 Jun 3;96(5):1660–74. doi: 10.1111/cdev.14259 (PMC12379839; doi:10.1111/cdev.14259)
Supplement: Supplementary file 1 — Data S1. [file CDEV-96-1660-s001.docx]

**Supplemental materials**

**Exploratory analyses**

***Study 1***

**Age.** Non-preregistered, exploratory analyses revealed a significant age × help type (direct, indirect) interaction, *B* = 0.84, SE = .39, *p* = .029, 95% CI [0.13, 1.74]. Simple slope analyses did not yield significant slopes (*ps* >.122). After receiving *indirect help*, younger children were less motivated, *B* = -0.62, SE = 0.28, *p* = .026, 95% CI [-1.31, -0.09]. After receiving *direct help*, however, age was not significantly related to motivation, *B* = -0.22, SE = 0.27, *p* = .405, 95% CI [-0.25, 0.73].

**Performance.** On average children provided 3.10 (out of the possible 6) correct answers (*SD* = 1.24). To examine whether children’s performance influenced the results, we repeated our analyses including a main effect an interaction with each dummy variable with the number of puzzles children answered correctly.

For task difficulty there was a significant interaction with performance and receiving no help vs. indirect help, *B* = 0.42, SE = 0.17, *p* = .013, 95% CI [0.09, 0.76]. Simple slope analyses showed that when children performed well they perceived the block test as equally difficult after receiving indirect vs. no help, *B* = 0.23, SE = 0.25, *p* = .351, 95% CI [-0.25, 0.71]. But when children performed less well, they perceived the block-test as easier after receiving no help compared to receiving indirect help, *B* = -0.62, SE = 0.23, *p* = .006, 95% CI [-1.06, -0.18].

For clarity of task instructions, there was a significant interaction of performance with direct vs. indirect help, *B* =-0.41, SE = 0.17, *p* = .016, 95% CI [-0.74, -0.08]. Simple slope analysis showed that when children performed well, they perceived instructions as equally clear after receiving indirect or direct help, *B* =-0.22, SE = 0.24, *p* = .350, 95% CI [0.35, -0.70]. And when children performed less well, they perceived the instructions as more clear when they received indirect help compared to direct help, *B* = -0.59, SE = 0.24, *p* = .013, 95% CI [0.13, 1.06].

***Study 2***

**Age.** For children’s general self-perceived ability, a significant interaction emerged for age and whether children received direct vs. indirect help, *B* = -0.39, SE = 0.19, *p* = .037, 95% CI [-0.76, -0.03]. Older children thought worse about their own abilities after receiving *indirect help* compared to *direct help*, *B* = -0.51, SE = 0.21, *p* = .002, 95% CI [-0.93, -0.09]. For young children there was no difference, *B* = 0.14, SE = 0.22, *p* = .544, 95% CI [-0.31, 0.58]. In addition, after receiving *direct help,* children’s age was not related to general self-perceived ability, *B* = 0.09, SE = 0.13, *p* = .510, 95% CI [-0.18, 0.35]. But after receiving *indirect help*, children reported lower self-perceived ability with age, *B* = -0.31, SE = 0.15, *p* = .046, 95% CI [-0.61, -0.01].

**Gender.** For perceived task difficulty there was a significant interaction for gender and receiving direct help vs. no help, *B* = 0.45, SE = 0.21, *p* = .029, 95% CI [0.05, 0.86]). However, for both girls and boys the comparison between conditions was not significant (*ps* > .091).

For persistence, there was a significant interaction for gender and type of help received (indirect vs. direct), *B* = -9.57, SE = 4.02, *p* = .018, 95% CI [-17.50, -1.64]. Boys persisted less after receiving direct help compared to indirect help, *B* = -14.23, SE = 5.63, *p* = .013, 95% CI [-25.43, -3.03] and for girls there was no difference between conditions, *B* = 4.92, SE = 5.73, *p* = .39, 95% CI [-6.46, 16.30].

***Study 3***

**Gender.** For challenge seeking there was a significant interaction for gender and receiving direct help vs. indirect help**,** *B* = 0.48, SE = 0.24, *p* = .047, 95% CI [0.01, 0.96]. Boys sought fewer challenges after receiving direct help compared to indirect help, *B* = 0.81, SE = 0.36, *p* = .012, 95% CI [0.09, 1.53], but there was no difference for girls**,** *B* = -0.16, SE = 0.32, *p* = .632, 95% CI [-0.80, 0.49].

**Performance.** The influence of children’s performance was assessed and two significant interactions emerged for perceived difficulty. First, there was a significant interaction for perceived task difficulty and receiving an indirect and direct help, *B* = 0.55, SE = 0.21, *p* = .010, 95% CI [0.13, 0.97]. Second, there was a significant interaction for perceived task difficulty and receiving indirect help and no help, *B* = 0.57, SE = 0.21, *p* = .007, 95% CI [0.16. 0.99]. Simple slope analyses showed that when children performed well, the type of help they received did not influence perceptions of difficulty (indirect vs. direct help, *B* = 0.52, SE = 0.30, *p* = .087, 95% CI [-0.08, 1.12]; indirect vs. no help, *B* = 0.39, SE = 0.29, *p* = .180, 95% CI [-0.18, 0.96]. But when children did not perform so well, children who received indirect help perceived the block-test as more difficult compared to children who received direct help, *B* = -0.58, SE = 0.28, *p* = .042, 95% CI [-1.14, -0.02] or no help, *B* = -0.75, SE = 0.30, *p* = .012, 95% CI [-1.14, -0.17].

In addition, a significant interaction emerges for self-perceived ability at the task and receiving indirect help and no help, *B* = -0.23, SE = 0.18, *p* = .023, 95% CI [0.60, 0.79]. Simple slope analysis showed that when children performed well, receiving help did not influence self-perceived ability *B* = 0.19, SE = 0.25, *p* = .457, 95% CI [-0.31, 0.69]. But when children performed less well, they felt worse about their ability at the task after receiving indirect help compared to no help, *B* = -0.66, SE = 0.26, *p* = .012, 95% CI [-1.17, -0.15].

**Pre-task general self-perceived ability.** For perceived task difficulty, there was a significant interaction for pre-task ability and indirect help vs. no help, *B* = -0.42, SE = -0.25, *p* = .026, 95% CI [-0.79, -0.05]. When children felt smart before taking the test, they perceived the block test as more difficult after receiving indirect help than no help *B* = -0.57, SE = 0.27, *p* = .039, 95% CI [-1.10, -0.03]. When children felt less smart before taking the test, they perceived the block test as equally difficult after receiving direct help and no help *B* = -0.07, SE = 0.30, *p* = .821, 95% CI [-0.66, 0.52].

For how much children liked the help that they received, there was also a significant interaction with pre-task ability and receiving direct help vs. no help *B* = -0.90, SE = 0.32, *p* = .005, 95% CI [-1.53, -0.27]. Simple slope analyses showed that when children felt smart, they disliked receiving direct help more than receiving no help *B* = -1.51, SE = 0.39, *p* < .001, 95% CI [-0.79, -0.05]. But when children felt less smart, they liked receiving direct help and no help equally *B* = 0.29, SE = 0.10, *p* = .510, 95% CI [-0.58, 1.15].

For persistence, there was a significant interaction for pre-task ability and receiving indirect help vs. no help *B* = 14.12, SE = 6.82, *p* = .040, 95% CI [27.58, 0.67]. When children felt smart before taking the test, they persisted longer after receiving no help compared to receiving indirect help *B* = 25.07, SE = 9.90, *p* = .012, 95% CI [5.53, 44.60]. But when children felt less smart before the test, they persisted equally longer after receiving indirect help and no help *B* = -3.18, SE = 9.70, *p* = .744, 95% CI [-22.33, 15.97].

***Study 4: Internal Meta-Analysis***

**Gender**. Children’s gender moderated how much children enjoyed the block test after receiving indirect help vs. no help, *B* = 0.25, SE = 0.10, *p* = .008, 95% CI [0.07, 0.44]. Follow-up analyses showed that girls who received indirect help liked the block test less compared to girls who received no help, *B* = -0.35, SE = 0.13, *p* = .008, 95% CI [-0.61, -0.09], but boys who received indirect help and no help did not differ in how much they liked the block test, *B* = 0.15, SE = 0.14, *p* = .272, 95% CI [-0.12, 0.42].

**Performance.** For perceived task difficulty, a significant interaction emerged for task performance and receiving indirect versus direct help, *B* = 0.24, SE = 0.11, *p* = .029, 95% CI [0.03, 0.46] and indirect versus no help, *B* = 0.25, SE = 0.11, *p* = .020, 95% CI [0.40, 0.47]**.** However, simple slope analyses showed that both when children performed well and when they performed less well, they perceived the block test as equally difficult after receiving direct and indirect help (high performance: *B* = 0.25, SE = 0.15, *p* = .099, 95% CI [ -0.05, 0.56]; low performance: *B* = -0.23, SE = 0.16, *p* = .149, 95% CI [-0.53, 0.08]).

For children performing well indirect help versus no help also did not matter for perceived task difficulty, *B* = 0.13, SE = 0.16, *p* = .423, 95% CI [-0.18, 0.43]. The sole significant slope that emerged was for children performing less well who received indirect help versus no help, *B* = -0.38, SE = 0.15, *p* = .011, 95% CI [-0.68, -0.09]: Children who did not do so well on the block test and received a hint, perceived the test as more difficult than those who received no help.

**Ethnicity.** We also examined how ethnicity (contrast coded: Dutch vs. not Dutch) influenced the results. There were no main effect of ethnicity and ethnicity did not moderate findings for task liking, task difficulty, self-perceived ability task and need (all p’s > .184)

**Household income**. There were no main effect of income and income did not moderate findings for task liking, task difficulty, self-perceived ability task and need (p’s > .097).

**Education parents.** Educational level of caretaker (mean score) moderated how difficulty children thought the task was when they received direct help versus no help, *B* = -0.24, SE = 0.11, *p* = .038, 95% CI [0.01, 0.46]. When caretakers had obtained a higher level of education (1 SD above the mean), children who received direct help thought the task was more difficult than children who received no help, *B* = 0.41, SE = 0.16, *p* = .011, 95% CI [0.09, 0.73]. When caretakers had obtained a lower level of education (1 SD below the mean), children who received direct help thought the task was equally difficult compared to children who received no help, *B* = -0.07, SE = 0.16, *p* = .687, 95% CI [-0.38, 0.25]. There were no other effects for education of caretakers.

**Table S1**

*Means and SD’s for Study 1*

|  | Study 1 | | |
| --- | --- | --- | --- |
|  | Direct help | Indirect help | No help |
| Task liking | 5.13 (1.06) | 5.56 (0.63) | 5.45 (0.92) |
| Task difficulty | 3.71 (0.95) | 3.85 (1.10) | 4.08 (1.12) |
| Task instructions | 5.00 (1.17) | 5.16 (1.07) | 5.26 (0.98) |
| Self- ability | 4.14 (1.14) | 4.31 (0.98) | 4.73 (1.06) |
| Self-effort | 5.65 (0.78) | 5.56 (0.76) | 5.62 (0.67) |
| Self-growth | 4.98 (1.20) | 4.93 (1.13) | 4.91 (1.27) |
| Motivation | 1.25 (0.43) | 1.25 (0.43) | 1.18 (0.38) |
| Need | 3.16 (1.22) | 2.94 (1.21) | 2.16 (1.05) |

**Table S2**

*Study 2 and 3 Means and SD’s*

|  | Study 2 | | | Study 3 | | |
| --- | --- | --- | --- | --- | --- | --- |
|  | Direct help | Indirect help | No help | Direct help | Indirect help | No help |
| Task liking | 5.37 (0.90) | 5.19 (1.21) | 5.36 (0.84) | 5.23 (0.91) | 5.31 (1.04) | 5.62 (0.64) |
| Task difficulty | 3.85 (1.18) | 3.75 (1.08) | 3.73 (1.22) | 3.98 (1.12) | 3.92 (1.34) | 4.10 (1.07) |
| Ability task | 4.29 (0.77) | 4.12 (1.13) | 4.24 (1.01) | 4.19 (1.12) | 4.24 (0.96) | 4.47 (0.89) |
| Challenge seeking | 1.95 (1.27) | 2.07 (1.31) | 2.02 (1.22) | 1.54 (1.24) | 1.59 (1.12) | 2.33 (1.11) |
| Persistence | 54.20 (20.36) | 58.81 (25.20) | 55.10 (18.19) | 56.28 (39.08) | 52.28 (19.98) | 63.42 (50.13) |
| Ability general | 5.19 (0.88) | 5.00 (1.01) | 5.19 (0.74) | 4.91 (1.03) | 5.01 (1.01) | 5.14 (0.78) |
| Like receiving help | 3.85 (1.57) | 4.74 (1.53) | 4.63 (1.26) | 4.27 (1.53) | 5.02 (1.26) | 4.98 (1.16) |
| Need | 2.90 (1.17) | 2.88 (1.29) | 2.27 (1.22) | 3.02 (1.26) | 3.07 (1.30) | 2.07 (1.02) |

Note. Means (SD) for persistence are without outlier, means (SD’s) for like receiving help are without those that misreported the type of help received.

| **Table S3**  *Ethnicities Reported by Parents when they Reported Their Child Was Not Dutch* | | | |
| --- | --- | --- | --- |
| *Ethnicity* | Study 1 | Study 2 | Study 3 |
| African | 1 |  |  |
| Antillian |  |  | 1 |
| Austrian |  |  | 1 |
| Azian |  | 1 |  |
| Belgium | 1 |  |  |
| Belt | 1 |  |  |
| British |  | 1 |  |
| Chinese | 2 |  |  |
| Cur-nel | 1 |  |  |
| Dutch-African |  | 2 |  |
| Dutch-American |  | 1 | 1 |
| Dutch-Belgium |  | 1 |  |
| Dutch-Bolivian |  | 1 |  |
| Dutch-Brazilian | 1 |  |  |
| Dutch-British |  | 2 |  |
| Dutch-Chinese |  |  | 1 |
| Dutch-Danish | 1 |  |  |
| Dutch-Dominican |  | 1 |  |
| Dutch-German |  | 1 | 1 |
| Dutch-Hindustani | 1 |  |  |
| Dutch-Italian | 3 |  |  |
| Dutch-Korean | 1 |  |  |
| Dutch-Moroccan | 2 |  |  |
| Dutch-Peruvian |  | 1 |  |
| Dutch-Portugese |  | 1 |  |
| Dutch-Spanish | 1 |  |  |
| Dutch-Surinamese | 5 |  |  |
| Dutch-Surinamese- Haitian- German |  | 1 |  |
| Dutch-Thai-Argentian | 2 |  |  |
| Dutch-Turkish | 6 |  |  |
| Dutch-Turkish-Bulgarian |  | 1 |  |
| Dutch-Yemenis |  | 1 |  |
| Flemish | 1 |  |  |
| German | 1 |  | 1 |
| Hindustani |  | 1 |  |
| Hindustani -Surinamese |  | 2 |  |
| Indonesian |  |  | 1 |
| Kaap-suri | 1 |  |  |
| Macedonia | 1 |  |  |
| Moroccan | 1 | 3 | 2 |
| Polish |  |  | 2 |
| Portugese |  |  | 1 |
| Romanian | 1 |  |  |
| Russian | 1 |  |  |
| Surinamese |  |  | 1 |
| Swiss |  |  | 1 |
| Turkish | 3 |  |  |

**Table S4**

*Changes in Context and Dependent Variables Across Studies*

|  | **Study 1** | **Study 2** | **Study 3** |
| --- | --- | --- | --- |
| Context | Science museum | Online | Science museum |
| Task-perceptions | Perceived task difficulty (1 item) ^a^  Task liking (1 item) ^a^  Clarity of instructions ^a^ | Perceived task difficulty (1 item) ^a^  Task liking (1 item) ^a^ | Perceived task difficulty (1 item) ^a^  Task liking (1 item) ^a^ |
| Self-perceptions | Task-specific self-perceived ability (1 item) ^a^  Self-perceived potential for growth (1 item) ^a^  Task-specific self-perceived effort (1 item) ^a^ | Task-specific self-perceived ability (3 items) ^a^  General self-perceived ability (3 items) ^a^ | Task-specific self-perceived ability (3 items) ^a^  General self-perceived ability (3 items) ^a^ |
| Task motivation | “Do you want to do another puzzle?” (1 item)^a^ | Persistence^a^  Challenge seeking ^a^ | Persistence ^a^  Challenge seeking ^a^ |
| Perceived need | “Did you need help with the block-test, you think?”^b^ | “Did you need help with the block-test, you think?”^b^ | “Did you need help with the block-test, you think?”^a^ |
| Self-reported type of help received | “What type of help did you receive for the last puzzle?”^b^ | “What type of help did you receive for the last puzzle?”^b^ | “What type of help did you receive for the last puzzle?”^a^ |
| Liking of help | Not included | “Did you like receiving no help/a hint/the correct answer on the last block test?”^b^ | “Did you like receiving no help/a hint/the correct answer on the last block test?”^a^ |
| Perceived performance | Not included | “How many puzzles did you think you solved before the computer told you it was 3?”^b^ | “How many puzzles did you think you solved before the computer told you it was 3?”^b^ |

*Note.* All variables were assessed only after the experimental manipulation, except general self-perceived ability in Study 3, which was assessed both before and after the task.

^a^Confirmatory (i.e., condition effects on this dependent variable were preregistered). ^b^Exploratory (i.e., condition effects on this dependent variable were not preregistered).

**Figure S1**

*Means and Standard Error Across the Three Studies (Study 4: Internal Meta-Analysis)*
